# The Neurotoxic Effect of Environmental Temperature Variation in Adult Zebrafish (*Danio rerio*)

**DOI:** 10.3390/ijms242115735

**Published:** 2023-10-29

**Authors:** Elisa Maffioli, Simona Nonnis, Francesca Grassi Scalvini, Armando Negri, Gabriella Tedeschi, Mattia Toni

**Affiliations:** 1 Department of Veterinary Medicine and Animal Science (DIVAS), Università degli Studi di Milano, Via dell’Università 6, 26900 Lodi, Italy; elisa.maffioli@unimi.it (E.M.); simona.nonnis@unimi.it (S.N.); francesca.grassiscalvini@unimi.it (F.G.S.); armando.negri@unimi.it (A.N.); 2 CRC “Innovation for Well-Being and Environment” (I-WE), Università degli Studi di Milano, 20126 Milano, Italy; 3 Department of Biology and Biotechnologies “Charles Darwin”, Sapienza University, Via Alfonso Borrelli 50, 00161 Rome, Italy

**Keywords:** neurotoxicity, temperature, zebrafish, BDNF, proteomic, behaviour

## Abstract

Neurotoxicity consists of the altered functionality of the nervous system caused by exposure to chemical agents or altered chemical–physical parameters. The neurotoxic effect can be evaluated from the molecular to the behavioural level. The zebrafish *Danio rerio* is a model organism used in many research fields, including ecotoxicology and neurotoxicology. Recent studies by our research group have demonstrated that the exposure of adult zebrafish to low (18 °C) or high (34 °C) temperatures alters their brain proteome and fish behaviour compared to control (26 °C). These results showed that thermal variation alters the functionality of the nervous system, suggesting a temperature-induced neurotoxic effect. To demonstrate that temperature variation can be counted among the factors that generate neurotoxicity, eight different protein datasets, previously published by our research group, were subjected to new analyses using an integrated proteomic approach by means of the Ingenuity Pathway Analysis (IPA) software (Release December 2022). The datasets consist of brain proteome analyses of wild type adult zebrafish kept at three different temperatures (18 °C, 26 °C, and 34 °C) for 4 days (acute) or 21 days (chronic treatment), and of BDNF^+/−^ and BDNF^−/−^ zebrafish kept at 26 °C or 34 °C for 21 days. The results (a) demonstrate that thermal alterations generate an effect that can be defined as neurotoxic (*p* value ≤ 0.05, activation Z score ≤ −2 or ≥2), (b) identify 16 proteins that can be used as hallmarks of the neurotoxic processes common to all the treatments applied and (c) provide three protein panels (*p* value ≤ 0.05) related to 18 °C, 34 °C, and BDNF depletion that can be linked to anxiety-like or boldness behaviour upon these treatments.

## 1. Introduction

Ongoing global warming has a profound effect on both freshwater and saltwater ecosystems, resulting not only in temperature shifts but also triggering a series of events, including the rapid melting of glaciers and ice [1,2], the rise in sea levels [3], and the increasingly frequent occurrence of cyclones and floods [4,5], which can impact the chemical–physical characteristics of the environment.

Temperature is a fundamental abiotic parameter for the survival of organisms, as cells can only live within a specific temperature range. Among vertebrates, environmental temperature variations affect homoeothermic and heterothermic organisms differently. The former have thermal homeostasis mechanisms that regulate the exposure of single cells to thermal variation [6], while the latter have body temperatures that depend on the ambient temperature [6,7]. Heterothermic organisms, such as fish, are therefore good models for studying the impact of temperature fluctuations on cells and the entire organism [8,9,10,11,12,13]. Of particular importance is the effect of thermal variations on neurons, highly specialized cells crucial for an organism’s survival.

Among teleost fishes, the zebrafish *Danio rerio* is a widely used model organism in various research fields including neuroanatomy, neurodevelopment, and neurobehavior [14,15], as well as in the context of eco- and neurotoxicology [16,17,18]. In fact, zebrafish offer numerous advantages for research [19,20]. They have transparent embryos, which allow for easy visualization of the developing nervous system. Their genetic manipulability enables researchers to create transgenic lines. They undergo rapid development, which allows their neural circuits to develop within a matter of days. Their high reproductive rate permits the production of numerous offspring, making them valuable for large-scale studies of toxic substances or genetic screens. Furthermore, zebrafish share homology with mammalian systems, as they possess many genetic and cellular similarities in their nervous systems when compared to mammals. Their suitability for studying the neural circuits involved in behaviours such as locomotion, learning, memory, and social interactions is enhanced by behavioural assays. Lastly, zebrafish are cost-effective in terms of maintenance. Zebrafish prove invaluable for studying the impact of thermal variations on the central nervous system [9,10,11,12].

Neurotoxicity is defined as “any adverse effect on the chemistry, structure or function of the nervous system, during development or at maturity, induced by chemical or physical influences” [21,22]. Consequently, the presence of water pollutants or thermal variation could potentially exert neurotoxic effects in fish. Neurotoxicity studies are complex and often necessitate a multidisciplinary approach that considers alterations in nervous system development, the expression of genes and proteins considered markers of neurotoxicity, and animal behaviour. The zebrafish is particularly well-suited for neurotoxicity studies as it enables research at all developmental stages, including embryos, larvae, and adults [20,23,24,25,26].

In recent years, our research group has conducted proteomic and behavioural studies on wild type (WT) adult zebrafish exposed to temperatures of 18 °C (low), 26 °C (control), or 34 °C (high temperature) for either 4 days (acute) or 21 days (chronic treatment) [9,10,11,12] to investigate the impact of thermal variations on the nervous system of zebrafish. The selection of these three temperature values was based on the research conducted by Vergauwen et al. [27], taking into consideration the thermal tolerance of the zebrafish, which ranges from 6.7 °C to 41.7 °C [28,29]. These temperatures were chosen to encompass a range of ±8 °C relative to the control temperature of 26 °C, with the aim of replicating conditions that zebrafish may encounter in their natural habitat. In the wild, zebrafish frequently experience daily temperature fluctuations ranging from 0.1 °C to 5.6 °C [30], as well as seasonal variations ranging from 6 °C in winter to over 38 °C in summer [31]. The findings from our prior studies have shown that fluctuation in temperature leads to a change in both brain proteome and behaviour [10,11,12], indicating a potential impairment in the functioning of the nervous system. Proteomic data revealed that several proteins involved in cytoskeletal organization, mitochondrial regulation, energy metabolism, synapse function, and neurotransmitter release are differently regulated at extreme temperatures [10,12]. Behavioural tests indicated altered locomotor activity and impaired cognitive abilities in zebrafish at both 18 °C and 34 °C, suggesting a correlation between proteomic changes and behavioural alterations. Moreover, zebrafish kept at 18 °C exhibited anxiety-like behaviours, while those at 34 °C showed reduced anxiety and increased boldness [10,11,12].

Among the markers of neurotoxicity, BDNF holds a pivotal role [26,32,33,34,35], as this neurotrophin exerts a significant and multifaceted influence on the nervous system. It affects several processes such as neurogenesis, neuronal differentiation, survival, growth, and plasticity [36,37,38,39,40,41], as well as influencing animal behaviour [42,43,44,45,46]. Experimental evidence suggests a one-to-one relationship between BDNF and stress factors, as stress conditions alter the expression of BDNF [47,48,49,50,51,52,53,54,55,56,57], both in teleosts and mammals, and changes in BDNF expression, in turn, impact the animal’s cognitive function and behaviour [42,43,44,45,46], thereby influencing its response to stress conditions. Consistently, in adult zebrafish, we observed that exposure to 34 °C for 21 days resulted in reduced BDNF gene expression and an alteration in behaviour [9]. These results indicate a correlation between temperature and BDNF changes. However, they do not demonstrate direct causation, as thermal variations induce the altered expression of numerous genes and proteins which in turn could influence the altered expression of BDNF. Using heterozygous BDNF^+/−^ (HT) and knockout BDNF^−/−^ (KO) zebrafish, we confirmed that the reduced or absent expression of this neurotrophin affects both brain neurochemistry and behaviour, and altered cellular and behavioural responses to thermal treatment [9,58].

In the present study, we performed a novel bioinformatic analysis on the eight proteomic datasets previously published by our research group [9,10,12,58] to investigate potential neurotoxicity resulting from thermal treatments. The datasets refer to the brain proteome of wild type (WT) adult zebrafish maintained at temperatures of 18 °C, 26 °C, and 34 °C for either 4 or 21 days, and BDNF^+/−^ and BDNF^−/−^ adult zebrafish maintained at temperatures of 26 °C or 34 °C for 21 days. The results provide support for the neurotoxic effect of temperature variation in adult zebrafish and the role of BDNF in neurotoxicity.

## 2. Results

### 2.1. Common Protein Networks Involved in the Neurotoxicity Induced by Thermal Stress and the Alteration of BDNF Expression

**Figure 1:**
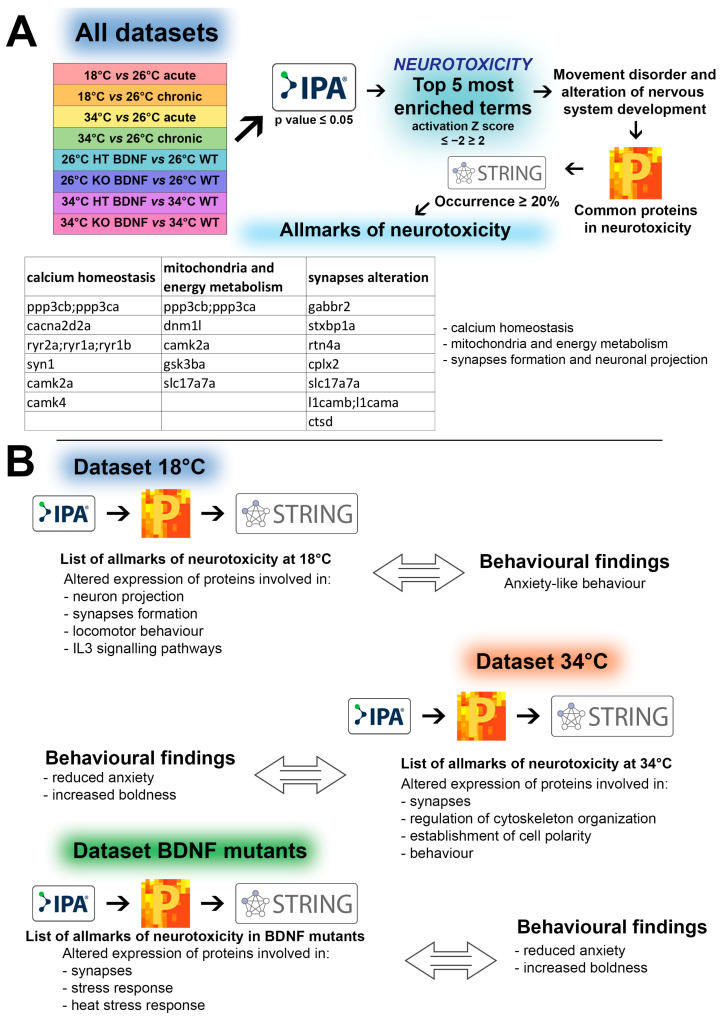
Workflow of the systematic analysis of the proteomic data. (**A**) Analysis conducted using IPA (significance level set at *p* ≤ 0.05) starting from the proteins showing differential expression in all datasets, reported in Tables S1–S8 adapted from [9,10,12]. (**B**) Analysis of the separate datasets of WT at 18 °C, WT at 34 °C, and BDNF mutants (HT and KO) at both 26 °C and at 34 °C. Experimental details are reported in the Section 4. All the lists of allmarks of neurotoxicity are reported in Table S12.

An integrated proteomic approach by means of the Ingenuity Pathway Analysis (IPA) was conducted based on the workflow described in Figure 1, obtaining the results reported below.

From our previous analyses [9,10,12], we extracted all the data listing the proteins that showed differential expression (whether increased, decreased, or exclusively expressed in a single condition) in the following eight comparisons, as detailed in Supplementary Tables S1–S8: WT_18 °C vs. WT_26 °C acute, WT_18 °C vs. WT_26 °C chronic, WT_34 °C vs. WT_26 °C acute, WT_34 °C vs. WT_26 °C chronic [10,12], HT BDNF_26 °C vs. WT_26 °C chronic, KO BDNF_26 °C vs. WT_26 °C chronic, HT BDNF_34 °C vs. WT_34 °C chronic, KO BDNF_34 °C vs. WT_34 °C chronic [9]. These datasets were previously generated using an identical label-free shotgun proteomic approach and the same statistical stringency as described in [9,10,12]. Although previously published, the lists of the proteins differentially expressed in the eight comparisons are reported in Supplementary Tables S1–S8 for reading convenience.

To provide context for the neurotoxicity analysis, whether resulting from thermal stress or alterations in BDNF expression, we conducted a new IPA Tox analysis on these datasets, employing a significance level of *p* ≤ 0.05 as detailed in the Section 4.

Considering the top five most enriched terms resulting from the analyses (Supplementary Table S9), it is evident that two major categories consistently emerge and are present in all datasets: neurological diseases and nervous system development, as summarised in Table 1.

In a few instances, specific networks related to these terms can also be identified: “cell to cell signalling and interaction, nervous system development and function, and cell morphology” in the WT_18 °C vs. WT_26 °C acute dataset; “neurological disease, organism injury and abnormalities, and tissue morphology” in the WT_18 °C vs. WT_26 °C chronic dataset; “cellular assembly and organization, nervous system development and function, infectious diseases” and “cell-to-cell signalling and interaction, cellular assembly and organization, and nervous system development and function” in the KO BDNF_34 °C vs. WT_34 °C dataset.

Among the top five most enriched terms found via IPA in each dataset, all results possibly related to neurotoxicity in terms of pathways, networks, and genes (shown in red in Supplementary Table S9) were manually extracted. These included terms such as neurological disease, nervous system development, and psychological disorders together with the corresponding subcategories. We focused specifically on terms with an activation Z score greater than 2 or less than −2, considering these values as significantly activated or inhibited, respectively (Supplementary Table S10 and Table 1). The results presented in Table 1 demonstrate that high temperature variation and chronic exposure to low temperature induce movement disorders and alteration of the nervous system development in zebrafish, similar to what is observed in the KO BDNF samples.

### 2.2. Common Proteins Involved in Temperature-Induced Neurotoxicity

To explore the presence of common proteins across various IPA Tox functions, pathways, and networks associated with neurotoxicity, we compared the proteins of the top five functions of all datasets with a significant Z score (Table 1 and Supplementary Table S10) using Perseus software (Version 1.6.1.40), looking for the occurrences of specific proteins. A total of 377 terms/categories were searched, allowing us to calculate the corresponding protein occurrence and to select those with a high occurrence (≥80/377, ≥20%) (Table 2).

**Figure 2:**
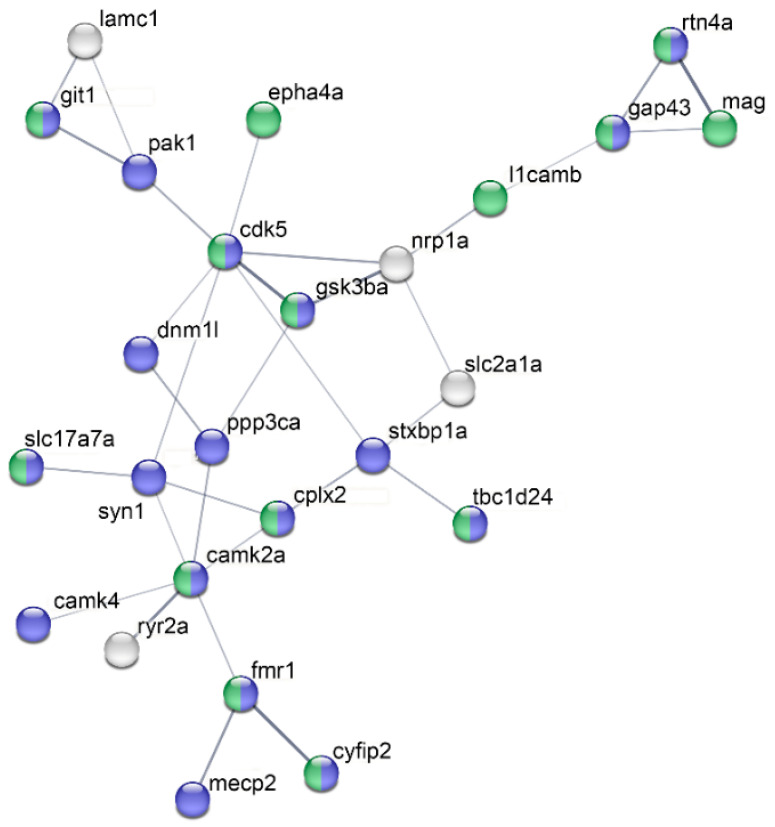
Protein association network analysis. The analysis was conducted using String for all the proteins listed in Table 2, employing a medium confidence threshold (0.4) for the minimum required interaction score. The thickness of the lines indicates the strength of the data support. For clarity, proteins not connected in the network are omitted. Following GO analysis, proteins involved in cell projection are indicated in green, while those related to synapse formation are depicted in blue.

We conducted a protein association network analysis on the proteins listed in Table 2 using String software (Version 11.5). The results showed a strong relationship among proteins regarding two cellular functions. This suggests that both thermal stress and the absence of BDNF expression induced a common pattern of neurotoxicity in zebrafish involving the impairment of synapse formation and neuronal projections (Figure 2).

The analysis of the neurotoxicity-related proteins common to all datasets listed in Table 2 reveals (a) the involvement of calcium homeostasis, (b) the entanglement of mitochondria and energy metabolism, and (c) impact at the synaptic level. This analysis has identified sixteen proteins that can serve as distinctive markers in the neurotoxic processes resulting from the various treatments (Table 1 and Table 3).

### 2.3. Neurotoxicity Associated with Anxiety-like or Boldness Behaviour

The main behavioural findings observed upon exposure to temperatures of 18 °C and 34 °C, previously described in our works [9,10,11,12], are summarized in Supplementary Table S11. The results clearly demonstrate two different profiles: anxiety-like behaviour exhibited by WT zebrafish at 18 °C (acute treatment), and reduced anxiety and increased boldness observed in WT zebrafish at 34 °C (chronic treatment). Interestingly, the latter profile closely resembles the behavioural patterns observed in KO zebrafish at both 26 °C and 34 °C, and, to some extent, in HT zebrafish. This similarity may be attributed to the reduced expression of the BDNF gene observed in WT zebrafish at high temperatures [9].

**Figure 3:**
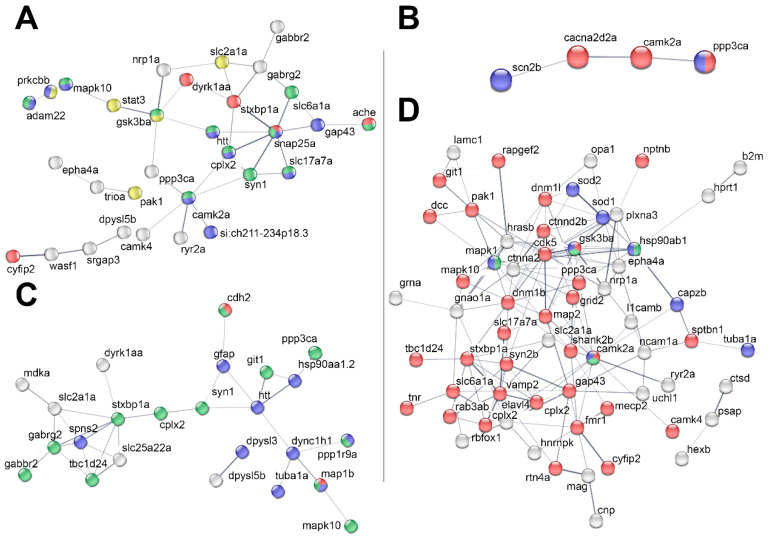
Protein association network analysis of 18 °C vs. 26 °C (**A**,**B**), 34 °C vs. 26 °C (**C**), and BDNF mutants (**D**). The analysis was conducted using String on the proteins listed in Table S12 with an occurrence ≥ 20%, using a medium confidence (0.4) for the minimum required interaction score. Line thickness indicates the strength of the data support. For clarity, the proteins not connected are not present in the figure. (**A**) 18 °C vs. 26 °C. Upon GO analysis, the proteins involved in neuron projections are indicated in blue, the proteins involved in synapse formation in green, proteins belonging to abnormal locomotory behaviour process quality are in red, and those to IL3 signalling pathways in yellow. (**B**) 18 °C vs. 26 °C, proteins of the “psychological disorder” category. Calcium signalling is indicated in red and the adaptive immune system network in blue. (**C**) 34 °C vs. 26 °C. In the figure, the following networks are indicated: synapse (green), regulation of cytoskeleton organization (blue), and establishment of cell polarity (red). (**D**) KO BDNF and HT BDNF at 26 °C and 34 °C vs. WT at the same temperature. In the figure the following networks are indicated: synapsis (red), stress response (blue), response to heat stress (green). Details on the timing of the observed alteration in specific protein abundance (chronic and/or acute) are reported in Table S12.

In an attempt to identify the main proteins involved in the two different behaviours, we followed the same methodology as detailed in the preceding section. We focused separately on the conditions of 18 °C and 34 °C, considering both acute and chronic exposure, as well as on HT and KO zebrafish at 26 °C and 34 °C. Specific protein occurrences were identified using the aforementioned approach and are documented in Table S12. Furthermore, a protein association network analysis was conducted using String (Figure 3 and Table 4).

In the comparison of WT_18 °C vs. WT_26 °C, the altered expression of proteins related to neuron projection (blue), synapse formation (green), locomotor behaviour process quality (red), and IL3 signalling pathways (yellow) was observed (Figure 3A,B and Table 4). These findings align with the behavioural data observed in zebrafish at 18 °C, which showed significant alteration in motor behaviour characterized by increased freezing events and a marked reduction in movement.

At 34 °C, String analyses revealed altered expression in protein networks associated with the synapse (green), regulation of cytoskeleton organization (blue), and establishment of cell polarity (red) (Figure 3C, Supplementary Table S12, and Table 4). 

Certain proteins, such as GABBR2, GABRG2, SYN1, HTT, PPP3CA, and MAPK10 displayed altered expression in both WT_18 °C vs. WT_26 °C and WT_34 °C vs. WT_26 °C comparisons, suggesting their potential contribution to the altered behaviour observed at both low and high temperatures. In HT BDNF and KO BDNF at 26 °C and 34 °C, the reduced or absent expression of BDNF led to profound changes in the brain proteome, affecting the expression of proteins primarily related to synapses, stress response, and heat stress response, as illustrated in Figure 3D, Supplementary Table S12, and Table 4. Notably, most of the proteins with the highest occurrence were associated with networks related to abnormal whole organisms, abnormal locomotion, and abnormal neurons according to the Zebrafish phenotype ontology in the String analysis. This underscores the essential role played by BDNF as a neurotransmitter and modulator of neuronal plasticity, crucial for learning and memory.

### 2.4. Upstream Regulators’ Analysis

Considering the observed changes in protein expression in the experimental datasets listed in Supplementary Tables S1–S8, we conducted an IPA upstream analysis to identify upstream regulators and predict whether they are activated or inhibited. This analysis aimed to unveil the mechanistic networks potentially involved. The results are reported in Table 5, which provides a list of the top five upstream regulators for each condition.

As expected, a significant number of the identified proteins are transcription regulators or are involved in gene expression control (indicated in italics in Table 5). This finding helps elucidate the drastic proteome changes observed in response to temperature stress.

At 18 °C and 34 °C, the analysis suggests numerous protein factors involved in the early stages of differentiation and chromatin remodelling as potential upstream regulators. While their primary functions are associated with transcriptional regulation, emerging evidence suggests their involvement in learning, memory abilities, and response to stress conditions [59,60].

The analysis of upstream regulators also reveals the altered expression of proteins that form intracellular aggregates under various stress conditions: HTT (Huntingtin), APP (amyloid Beta precursor), and MAPT (microtubule associated protein tau) [61,62,63].

However, as shown in Table 5, only a few of these protein upstream regulators are predicted to be activated or inhibited, and only at high temperatures (34 °C): NFE2L2 is inhibited after long-term exposure, and CLPP and TP53 are activated in the BDNF mutants.

The other two upstream regulators with a significant Z score are CD 437, which is activated in KO BDNF mutants at 26 °C, and beta-estradiol, which is inhibited in HT BDNF mutants at the same temperature.

In summary, our data suggest that exposure to either low or high temperature, or the diminished or absent expression of BDNF, triggers a cellular response akin to that observed in cases of oxidative stress involving mitochondria.

This leads to an increase in the Tox functions such as seizure at both temperature extremes, alongside a reduction in Tox functions like coordination at 34 °C. These alterations give rise to entirely contrasting animal behaviours due to the expression of different proteins at 18 °C and 34 °C.

## 3. Discussion

The current study provides support for the neurotoxic effect of temperature variation in adult zebrafish. It demonstrates alterations in the expression of neurotoxicity-related proteins in the brains of experimental subjects exposed to temperatures of 18 °C and 34 °C for 4 days. Importantly, these alterations in neurotoxicity-related protein expression persist even after a 21-day thermal treatment. This suggests that the organism does not activate adaptive mechanisms to mitigate the neurotoxic effect after a period of three weeks. This is further corroborated by the persistence of the altered behaviour observed under the same conditions. 

The key findings of our proteomic and behavioural analysis indicate that exposure to low temperature (18 °C) results in alterations in protein expression associated with an increase in significant categories, including movement disorders, neurodegeneration in the brain, and abnormalities in the cerebral cortex, even upon acute exposure. This is accompanied by an increase in the protein pathways associated with memory, coordination, and synapse potentiation, possibly as an adaptive response to cope with the thermal stress. Chronic exposure to 18 °C consolidates the pathological framework, leading to the altered expression of Tox function-related proteins with a significant rise in generalized seizure and decreased coordination (Table 1 and Supplementary Table S9).

Similar effects were observed at 34 °C, where acute treatment notably affects proteins involved in morphogenesis and neuritogenesis, while promoting those involved in the transport of synaptic vesicles and endocytosis, processes also influenced by acute exposure to lower temperatures. Chronic exposure to 34 °C resulted in an increase in protein pathways associated with neuronal degeneration, seizures, and impaired coordination. These results suggest that the reduced expression of proteins associated with morphogenesis and neuritogenesis observed during acute exposure to 34 °C ultimately leads to neuronal degeneration, seizure, and myoclonus in the chronic condition. The comparison between the results of chronic and acute treatments reveals that the neurotoxic effects of thermal alterations do not diminish over time during the initial three weeks; instead, they intensify. This suggests that within the adult zebrafish brain, there are no evident acclimatization or adaptation mechanisms to mitigate the neurotoxic effects induced by temperature changes, even after 21 days. This hints at the possibility that extending the exposure to thermal variations may further exacerbate the neurotoxic effect (Table 1 and Supplementary Table S9).

Particularly noteworthy is our research group’s observation that subjecting zebrafish to elevated temperatures leads to a decrease in BDNF gene expression and that the reduced or absent expression of BDNF in adult mutant zebrafish induced altered behaviour [9], underscoring the connection between stress, BDNF expression, and behaviour.

The availability of the brain proteome from BDNF^+/−^ and BDNF^−/−^ zebrafish maintained at temperatures of 26 °C or 34 °C for 21 days [9] has enabled us to explore the neurotoxic effects resulting from the reduced or absent expression of this neurotrophin in zebrafish, as well as its role in the response to heat treatment. Reduced BDNF expression in HT zebrafish results in alterations in the brain proteome, leading to an increase in movement disorder categories. In contrast, its absence in KO zebrafish has a more profound impact, resulting in a decreased protein network, categories, Tox functions such as neuronal cell proliferation, neurite growth, and synapse development, along with increased motor dysfunction, seizures, ataxia, and reduced coordination. 

The effects of reduced BDNF expression are notably exacerbated at 34 °C, where the impact on the expression of proteins in the neurological disorders’ category is associated with a significant decrease in proteins belonging to the categories of synaptic activity and impaired nervous system development and functions. Results demonstrate that the neurotoxic effect is more pronounced in KO zebrafish than in HT zebrafish, suggesting that the partial expression of BDNF in HT is sufficient to partially guarantee neuroprotective functions (Table 1 and Supplementary Table S9).

Collectively, these findings confirm a neurotoxic effect induced by thermal stress, thereby suggesting that temperature variation can indeed be considered one of the factors that contribute to neurotoxicity in zebrafish, comparable to the neurotoxicity induced by the lack of BDNF expression.

### 3.1. Common Proteins Involved in Temperature-Induced Neurotoxicity

The results also indicate that the tested conditions (temperature and BDNF expression) lead to alterations in common neurotoxicity-related protein pathways, affecting (a) calcium homeostasis, (b) mitochondria and energy metabolism, and (c) the formation and functioning of synapses involving proteins that can be considered hallmarks of neurotoxicity under different conditions (Table 2 and Table 3). Consistent with our findings, the existing literature supports the data confirming the involvement of the genes listed in Table 3 in mechanisms of toxicity, affirming their role as markers of neurotoxicity in zebrafish.

In detail:

(a) CACNA2D2 and RYR2 are channels that play a role in regulating calcium current density [64,65]. They are also associated with the activity-dependent structural plasticity of dendritic spines, neuronal cell death, hyperactive behaviours, and impaired learning and memory [66,67]. CACNA2D2 has been reported to be involved in methamphetamine-induced neurotoxicity [68], and RYRs in neurotoxicity induced by lead and 6-OHDA [69,70].

SYN1 serves as a regulator of synaptic vesicle trafficking [71,72]. It plays a role in controlling neurotransmitter release at the pre-synaptic terminal and in the regulation of axon outgrowth and synaptogenesis [73,74]. Moreover, it is involved in neurotoxic processes [75,76].

CAMK4 and CAMK2A are implicated in synapse plasticity, spatial learning, memory consolidation, and long-term potentiation [77]. CAMK2 is associated with neurotoxicity induced by alcohol [78] and other conditions [79,80,81], while CAMK4 is implicated in arsenic-induced neurotoxicity.

PPP3CA, the protein with the highest occurrence, involved in 169 out of 377 terms/categories, plays a crucial role in Ca^2+^-mediated signal transduction [82,83,84,85,86]. It is also involved in numerous other processes, including the calcineurin-NFAT signalling cascade, which is important in axonal growth and guidance during vertebrate development and mitochondrial processes [87,88]. PPP3CA is involved in the signalling mediated by neurotoxins such as prion-like proteins [89].

(b) The results provide support for the involvement of mitochondria and energy metabolism in the neurotoxicity induced by thermal stress or a lack of BDNF expression. PPP3CA induces DNM1L translocation to the mitochondrion in response to increased Ca^2+^ levels following mitochondrial depolarization. Therefore, DNM1L is involved in neurotoxic events affecting mitochondria [90,91].

Accordingly, PPP3CA is directly associated with DNM1L and CAMK2A in the String analysis depicted in Figure 2. Notably, DNM1L and CAMK2A are among the most frequently occurring proteins (126 and 128, respectively) (Table 2).

GSK3B, CAMK2A, and PPP3CA are components of the Wnt signalling pathway, which was significantly affected by thermal stress under our experimental conditions. In line with this, recent findings by Xu et al. [92] demonstrate the strong implication of the Wnt/β-Catenin signalling pathway in cadmium-induced developmental neurotoxicity and neuroinflammation. GSK3B is involved in neurotoxicity induced by kainic acid [93] and FUS [94] and in TDP-43-mediated neurotoxicity [95].

The downregulation of SLC2A1 (solute carrier family 2 member 1) suggests an impact on energy metabolism, given that this protein is a critical energy carrier in the brain. It serves as a facilitative glucose transporter responsible for basal glucose uptake and is found at the blood–brain barrier [96,97,98,99,100]. SLCs are linked to neurotoxicity and neurodegeneration phenomena [101]; in particular, SLC2A1 is associated with neurotoxicity induced by tetrabromobisphenol A-bis (2-hydroxyethyl) ether [102].

(c) The results of the proteomic analysis also indicate that neurotoxicity affects the synaptic function in our model. STXBP1, CPLX1, and L1CAM (CHL1) are differentially expressed proteins involved in the transport, docking, and membrane fusion of synaptic vesicles. CPLX1 is involved in neurotoxic events associated with alpha-synuclein [103] and AβPP [104] pathologies and L1CAM in ethanol-induced developmental neurotoxicity [105].

The reduced expression of GABBR2 could have implications for neurons, as it plays a crucial role in the formation of functional inhibitory GABAergic synapses [106] and is involved in the neurotoxic action of vincristine [107]. The STXBP1 protein is involved in neurotransmitter release by regulating syntaxin, a transmembrane attachment protein receptor. In zebrafish, mutations in this gene are associated with epilepsy and behavioural abnormalities [108]. RTN4 regulates neurite fasciculation, branching and extension in the developing nervous system, and contributes to plasticity in the adult CNS [109,110].

The reduction in CPLX1 observed in our proteomic analysis could lead to impairment in synaptic vesicle trafficking and alterations in synaptic plasticity [111,112]. SLC17A7 is a vesicle-bound, sodium-dependent phosphate transporter primarily associated with synaptic vesicle membranes, and functions in glutamate transport [113].

CHL1 is the second most abundant protein listed in Table 2 (132 occurrences, 35%). It plays a critical role in various processes during brain development, including neuronal migration, axonal growth, and synaptogenesis [114]. CTSD plays a significant role in maintaining neuronal cell homeostasis through the proteolytic degradation of unfolded or oxidized protein aggregates that are delivered to lysosomes via autophagy or endocytosis [115]. Several neuronal proteins considered hallmarks of neurodegenerative diseases, such as the amyloid precursor, α-synuclein, and huntingtin, are physiologic substrates of CTSD. Their abnormal accumulation can occur if not efficiently degraded by this enzyme [116]. CTSD is involved in neurotoxicity phenomena [117,118], including tau-induced neurotoxicity [119].

### 3.2. Analysis of Upstream Regulators

Among the proteins modulated in the analysed datasets, there are also protein upstream regulators. Among these, NFE2L2 is inhibited in chronic exposure to 34 °C. It is a transcriptional regulator that coordinates the basal and stress-inducible activation of a wide array of cytoprotective genes [120]. In addition to this role, NRF2 has been shown to regulate mitochondrial bioenergetics and is involved in the unfolded protein response (UPR), which is triggered by the accumulation of misfolded proteins in the ER lumen.

Elevated temperature in BDNF mutants, instead, activated CLPP and TP53. CLPP (Caseinolytic Mitochondrial Matrix Peptidase Proteolytic Subunit) is a component of the CLP protease complex (Endopeptidase CLP), which plays a significant role in degrading misfolded proteins and participates in the final steps of RseA-sigma-E degradation, releasing sigma-E to induce the extra-cytoplasmic-stress response [121,122].

P53 is primarily known for its role as a tumour suppressor gene and its involvement in cancer development. However, it also plays essential functions in the brain, as it is involved in anxiety- and depression-like behaviours in mice [123] as well as behavioural traits and psychiatric disorders in humans [124].

CD 437, an antitumour toxin that induces cell apoptosis, is activated in KO BDNF mutants at 26 °C. Its activation in KO BDNF mutants may be linked to the anti-apoptotic role played by BDNF in protecting against neurodegeneration [125]. At the same temperature, beta-estradiol is inhibited in HT BDNF mutants. The inhibition of beta-estradiol may be related to the role of oestrogens in neurodegenerative diseases, which often overlaps with that observed for BDNF [126].

The analysis of upstream regulators also reveals the altered expression of proteins that form intracellular aggregates under various stress conditions: HTT (Huntingtin), APP (amyloid Beta precursor), and MAPT (microtubule-associated protein tau) [61,62,63]. All of them are also involved in oxidative stress damage, highlighting the influence of temperature variation on thermal and oxidative stress responses in fish, consistent with previous descriptions [127,128]. According to this analysis, the potent antioxidant 1,2-dithiol-3-thione is predicted to be inhibited upon chronic exposure at 34 °C.

### 3.3. Neurotoxicity Associated with Anxiety-like or Boldness Behaviour

Focusing on the neurotoxicity-associated anxiety-like or boldness behaviour, we have identified three panels of proteins related to these behaviours, as depicted in Figure 3 and listed in Table 4, along with Supplementary Table S12. These findings align with the behavioural alterations observed in vivo in WT at 18 °C, WT at 34 °C. and in BDNF mutants. They also pinpoint proteins worthy of investigation in future studies to elucidate their roles in anxiety-like and boldness behaviours.

Moreover, the results hold ecological significance, as the temperatures tested fall within the thermal tolerance range of the zebrafish and correspond to values observed in their natural habitat. Given the potential for global warming to exacerbate temperature fluctuations in the near future, these findings gain particular relevance. The neurotoxic impact of temperature is instrumental in driving behavioural changes that may have consequences for the survival of zebrafish. These alterations could render them more susceptible to predation or reduce their inclination to seek nourishment. Furthermore, the neurotoxic effects induced by temperature could compound those resulting from pollutants already present in the water. Consequently, the thermal fluctuations occurring in the natural environment, including those resulting from global warming, may heighten the risk of neurotoxicity in fish populations by amplifying the toxic impact of pollutants, even when present in low concentrations.

## 4. Materials and Methods

### 4.1. Thermal Treatment and Behavioural Analysis Performed in Previous Studies

No animal experiments were conducted in this study, as it primarily involves bioinformatic analysis of data obtained from online databases related to experiments conducted on zebrafish. The proteomics data used in this analysis are derived from three studies from our research group that employed similar housing procedures. For detailed information on the procedures, please refer to the respective works [9,10,12]. In summary, zebrafish were placed in tanks measuring 40 cm × 30 cm × 30 cm (width × depth × height) (referred to as “hometanks”) at a density of 1 zebrafish per litre. Initially, the zebrafish were acclimated to the tanks at a control temperature of 26 ± 1 °C during an adaptation period. To ensure good water quality, reverse osmosis pumps were used for water production, and a constant flow of filtered water (600 L/h) was maintained through external filter systems in each tank. Additionally, water was continuously aerated by aerators designed for aquaria (3300 cc/min, 200 L/h). The zebrafish were fed with commercial dry granular food. Water quality parameters, including water hardness, pH, ammonium (NH_4_), ammonia (NH_3_), nitrate (NO_3_), nitrite (NO_2_), phosphate (PO_4_), copper (Cu), and calcium (Ca^2+^), were periodically measured using specific kits. Faeces and remaining food waste were removed from the tanks at least three times per week, and during tank-cleaning procedures, approximately 20–30% of the water volume was exchanged to maintain the correct water volume and chemical–physical parameters.

The tanks that were dedicated to the thermal treatment were chosen randomly, and the water temperature was gradually adjusted, decreasing from 26 to 18 °C and increasing from 26 to 34 °C to initiate the thermal treatment. Zebrafish were then maintained at temperatures of 18 ± 1 °C and 34 ±  1 °C for either 4 days in the acute treatment or 21 days in the chronic treatment, while the control group of fish was kept at 26 ± 1 °C. At the conclusion of the thermal treatment, depending on the specific experiment, individuals underwent behavioural tests or were euthanized through prolonged immersion in a solution containing the anaesthetic tricaine methanesulfonate MS-222 (300 mg/L). Following euthanasia, the fish were decapitated, and their brains were dissected for subsequent proteomic analysis.

Depending on the work considered, following the completion of the thermal treatment, the experimental subjects were exposed to a range of behavioural tests such as Y-Maze, novel tank diving test, light and dark preference test, social preference test, and mirror biting test. All tests were conducted using water maintained at the same temperature as the thermal treatment administered. The tests were video-recorded and subsequently analysed using dedicated software such as Any-maze. During the video analysis, the experimenters conducting the assessments were unaware of the experimental group each subject belonged to. For further details, please consult the original publications.

### 4.2. IPA Tox Analysis

The analysis was conducted on proteins that exhibited differential expression (either increased, decreased, or exclusively expressed in one particular condition) in the following eight comparisons, which have been previously published in our earlier papers: WT_18 °C vs. WT_26 °C acute, WT_18 °C vs. WT_26 °C chronic, WT_34 °C vs. WT_26 °C acute, WT_34 °C vs. WT_26 °C chronic [10,12], HT BDNF_26 °C vs. WT_26 °C chronic, KO BDNF_26 °C vs. WT_26 °C chronic, HT BDNF_34 °C vs. WT_34 °C chronic, and KO BDNF_34 °C vs. WT_34 °C chronic [9].

All datasets were obtained using an identical label-free shotgun proteomic approach and adhered to the same statistical stringency as reported in [9,10,12]. In all cases, a database search was conducted against the zebrafish UniProt sequence database using the MaxQuant software; quantification was performed using the built-in XIC-based label-free quantification (LFQ) algorithm, and statistical analysis was carried out using the Perseus software.

In pair-to-pair comparisons, proteins were considered differentially expressed if they were present exclusively in one condition or showed a significant *t*-test difference (Welch’s test *p* ≤ 0.05), with 26 °C as the control. Although previously published, the lists of the proteins differentially expressed in the eight comparisons mentioned above are provided in Supplementary Tables S1–S8 for the sake of readability and adapted from the data reported in [9,10,12].

The IPA Tox analysis was conducted using the Ingenuity Pathway Analysis (IPA) software (Release December 2022). In detail: the proteins that exhibited differential expression in the eight pair-to-pair comparisons were loaded on the IPA along with the corresponding Welch’s *t*-test difference LFQ values, which were positive for the proteins more expressed at 18 °C or 34 °C, and negative for the proteins less expressed in comparison to the 26 °C control. For proteins exclusively expressed in one condition, the highest value, either positive or negative, was assigned. IPA Tox analysis was performed with a significance level set at *p* ≤ 0.05. If not otherwise indicated, *p*-values were calculated using the right-tailed Fisher’s exact test. The analysis considers only molecules and/or relationships that were either experimentally observed or had high-confidence predictions. It included only terms that presented an activation Z score, which is the summary value predicting activation (positive value) or inhibition (negative value) of a canonical pathway, upstream regulator, or downstream function, based on the gene expression changes within the network.

This approach was used because in IPA analysis neither *p*-value nor FDR account for whether genes are up-regulated or down-regulated in the dataset. Instead, the Z score activation predictions indicate the correspondence between the expected directional relationship and the observed gene expression in the dataset. Z scores greater than 2 or less than −2 were considered significantly activated or inhibited, respectively.

The occurrences of specific proteins were calculated using Perseus software (Version 1.6.1.40) [129]. The protein association network analysis was conducted using String (Version 11.5) [130] with a medium confidence threshold of 0.4 for the minimum required interaction score. Line thickness indicates the strength of the data support in the network analysis. Active interaction sources included experiments, text mining, databases, co-expression, neighbourhood, gene fusion, and co-occurrence.

## 5. Conclusions

The fluctuation in water temperature observed in the natural environment can exert a neurotoxic impact on zebrafish, even when it remains within the animal’s thermal tolerance range. This implies that thermal variations can potentially amplify the neurotoxic effects of pollutants that are already present in the environment. Additionally, these results reaffirm the crucial role of BDNF, as they underscore the neurotoxic effect resulting from the diminished or absent expression of this neurotrophin in zebrafish.

## Figures and Tables

**Table 1:** Summary of the major findings from the IPA Tox analysis. The IPA Tox analysis was performed using the Ingenuity Pathway Analysis (IPA) software (Release December 2022). The proteins showing differential expression in the eight pair-to-pair comparisons were loaded on IPA along with the corresponding Welch’s *t*-test difference LFQ values (positive values indicated proteins with higher expression at 18 °C, 34 °C, or in the BDNF mutants, while negative values indicated proteins with lower expression compared to the control at 26 °C or in WT). The IPA Tox analysis employed a significance level of *p* ≤ 0.05. The analysis considered only molecules and/or relationships experimentally observed or predicted with high confidence, as well as terms with a significant Z score (Z ≥ 2 for activation, represented by up arrows, and Z ≤ 2 for inhibition, represented by down arrows). “X” indicates the presence of the term/category in the specific dataset. (A) acute treatment, (C) chronic treatment. KO, BDNF^−/−^; HT, BDNF^+/−^.

	Tox Functions Related to Neurotoxicity (*p* Value ≤ 0.05) (From Table S9)								
		18 °C vs. 26 °C (A)	18 °C vs. 26 °C (C)	34 °C vs. 26 °C (A)	34 °C vs. 26 °C (C)	KO26 °C vs. WT26 °C	HT26 °C vs. WT26 °C	KO34 °C vs. WT34 °C	HT34 °C vs. WT34 °C
	Neurological Disease	X	X	X	X	X	X	X	X
	Nervous System Development and Function	X	X	X	X	X	X	X	X
	Psychological Disorders	X						X	
	**Functions related to neurological disease and nervous system development with a significant activation, Z score (From Table S10)**								
**Movement disorders**	Generalized seizures, environmental induced seizure, tonic-clonic seizure		**↑**		**↑**	**↑**		**↑**	**↑**
	Coordination		**↓**		**↓**	**↓**		**↓**	
	Movement Disorders, ataxia, convulsion					**↑**	**↑**	**↑**	
	Myoclonus				**↑**				
**Alteration of nervous system development**	MorphoGenesis of nervous tissue, synapse formation			**↓**		**↓**		**↓**	
	NeuritoGenesis, growth of neurites, neuronal cells proliferation			**↓**		**↓**		**↓**	
	Transport of synaptic vesicles			**↑**					
	Endocytosis of synaptic vesicles			**↑**				**↓**	
	Degeneration of neurons, encephalopathy				**↑**			**↑**	
	Brain tumour					**↓**			
	Brain lesion					**↓**			
**Common hallmarks of neurotoxicity induced by thermal stress and alteration of BDNF expression**									
Alteration of synapse formation and neuronal projections		X	X	X	X	X	X	X	X
Alteration of calcium homeostasis		X	X	X	X	X	X	X	X
Alteration of mitochondria and energy metabolism		X	X	X	X	X	X	X	X

**Table 2:** List of the proteins involved in the top five functions common to all datasets with a significant Z score and occurrence ≥ 20%. The table lists the proteins common in all datasets that exhibited high occurrence in the terms/categories related to neurotoxicity, as identified via IPA Tox analysis (≥80 out of 377 categories).

Gene (Human)	Gene Symbol (*Danio rerio*)	Ensembl Gene ID (*Danio rerio*)	Protein Name	Occurrence in 377 Terms/Categories	% Occurrence
*PPP3CA*	ppp3cb	ENSDARG00000025106	protein phosphatase 3 catalytic subunit alpha	169	45
	ppp3ca	ENSDARG00000004988			
*L1CAM*	l1camb	ENSDARG00000015025	cell adhesion molecule L1 Like	132	35
	l1cama	ENSDARG00000007149			
*CAMK2A*	camk2a	ENSDARG00000053617	calcium/calmodulin dependent protein kinase II alpha	128	34
*CACNA2D2*	cacna2d2a	ENSDARG00000103390	calcium voltage-gated channel auxiliary subunit alpha 2 delta 2	127	34
*DNM1L*	dnm1l	ENSDARG00000015006	dynamin 1 Like	126	33
*SLC2A1*	slc2a1b	ENSDARG00000007412	solute carrier family 2 member 1	120	32
	slc2a1a	ENSDARG00000001437			
*CDK5*	cdk5	ENSDARG00000056683	cyclin dependent kinase 5	119	32
*TBC1D24*	tbc1d24	ENSDARG00000069339	TBC1 domain family member 24	119	32
*GABBR2*	gabbr2	ENSDARG00000061042	Gamma-aminobutyric acid Type B Receptor	112	30
*MECP2*	mecp2	ENSDARG00000014218	methyl-CpG binding protein 2	111	29
*MAPK10*	mapk10	ENSDARG00000102730	mitogen-activated protein kinase 10	110	29
*GSK3B*	gsk3ba	ENSDARG00000017803	glycogen synthase kinase-3 beta	109	29
*C3*	c3a.1	ENSDARG00000012694	complement component 3	108	29
	c3a.2	ENSDARG00000087359			
*GNAD1*			G protein alpha stimulating activity polypeptide 1	108	29
*STXBP1*	stxbp1a	ENSDARG00000001994	syntaxin binding protein 1	107	28
*MAG*	Mag	ENSDARG00000104023	myelin associated glycoprotein	105	28
*PAK1*	pak1	ENSDARG00000103959	serine/threonine-protein kinase PAK 1	104	28
*CYFIP2*	cyfip2	ENSDARG00000036375	cytoplasmic FMR1 interacting protein 2	99	26
*GRN*	Grna	ENSDARG00000004954	granulin precursor	97	26
*LGI1*	lgi1a	ENSDARG00000020493	leucine rich glioma inactivated 1	97	26
*EPHA4*	epha4a	ENSDARG00000054454	EPH Receptor A4)	91	24
*GIT1*	git1	ENSDARG00000039489	ARF GTPase-activating protein	91	24
*RYR2*	ryr2a	ENSDARG00000098856	ryanodine receptor 2	90	24
	ryr1a	ENSDARG00000011422			
	ryr1b	ENSDARG00000023797			
*TUBA1A*	tuba1a	ENSDARG00000001889	tubulin Alpha 1a	89	24
*CTSD*	Ctsd	ENSDARG00000057698	cathepsin D	88	23
*LAMC1*	lamc1	ENSDARG00000036279	laminin subunit gamma 1	87	23
*SLC17A7*	slc17a7a	ENSDARG00000016480	solute carrier family 17 member 7	87	23
*FAM107A*	si:ch211-236d3.4	ENSDARG00000086300	family with sequence similarity 107 member A	85	23
*CAMK4*	camk4	ENSDARG00000005372	calcium/calmodulin-dependent protein kinase type IV	84	22
*GAP43*	gap43	ENSDARG00000099744	growth associated protein 43	84	22
*SYN1*	syn1	ENSDARG00000060368	synapsin 1	84	22
*FMR1*	fmr1	ENSDARG00000037433	fragile X messenger ribonucleoprotein 1	83	22
*NRP1*	nrp1a	ENSDARG00000102153	neuropilin 1	82	22
*RTN4*	rtn4a	ENSDARG00000112237	reticulon 4	81	21
*CPLX1*	cplx2	ENSDARG00000111920	complexin 1	80	21

**Table 3:** Hallmarks of neurotoxicity induced by thermal stress and the alteration of BDNF expression. The analysis of the proteins common to all the datasets in Table 2 shows their involvement in (a) calcium homeostasis, (b) mitochondria and energy metabolism, and (c) the formation and functioning of synapses.

Calcium Homeostasis			
Gene (Human)	Gene Symbol (*Danio rerio*)	Ensembl Gene ID (*Danio rerio*)	Protein Name
*PPP3CA*	ppp3cb	ENSDARG00000025106	protein phosphatase 3 catalytic subunit alpha
	ppp3ca	ENSDARG00000004988	
*CACNA2D2*	cacna2d2a	ENSDARG00000103390	calcium voltage-gated channel auxiliary subunit alpha 2 delta 2
*RYR2*	ryr2a	ENSDARG00000098856	ryanodine receptor 2
	ryr1a	ENSDARG00000011422	
	ryr1b	ENSDARG00000023797	
*SYN1*	syn1	ENSDARG00000060368	synapsin 1
*CAMK2A*	camk2a	ENSDARG00000053617	calcium/calmodulin dependent protein kinase II alpha
*CAMK4*	camk4	ENSDARG00000005372	calcium/calmodulin dependent protein kinase IV
Mitochondria and energy metabolism			
*PPP3CA*	ppp3cb	ENSDARG00000025106	protein phosphatase 3 catalytic subunit alpha
	ppp3ca	ENSDARG00000004988	
*DNM1L*	dnm1l	ENSDARG00000015006	dynamin 1 Like
*CAMK2A*	camk2a	ENSDARG00000053617	calcium/calmodulin dependent protein kinase II alpha
*GSK3B*	gsk3ba	ENSDARG00000017803	glycogen synthase kinase-3 beta
*SLC2A1*	slc17a7a	ENSDARG00000016480	solute carrier family 2 member 1
Synapses alteration			
*GABBR2*	gabbr2	ENSDARG00000061042	Gamma-aminobutyric acid Type B Receptor
*STXBP1*	stxbp1a	ENSDARG00000001994	syntaxin binding protein 1
*RTN4*	rtn4a	ENSDARG00000112237	reticulon 4
*CPLX1*	cplx2	ENSDARG00000111920	complexin 1
*SLC17A7*	slc17a7a	ENSDARG00000016480	solute carrier family 17 member 7
*L1CAM (CHL1)*	l1camb	ENSDARG00000015025	cell adhesion molecule L1 Like
	l1cama	ENSDARG00000007149	
*CTSD*	ctsd	ENSDARG00000057698	cathepsin D

**Table 4:** Protein association network analysis of 18 °C vs. 26 °C, 34 °C vs. 26 °C, and BDNF mutants. The table presents the genes of the networks shown in Figure 3. Green and red arrows indicate proteins with decreased or increased abundance, respectively. Detailed information regarding the timing of observed alterations in specific protein abundance (chronic and/or acute) is provided in Table S12.

18 °C vs. 26 °C	
Networks	Molecules
Neuron projections	adam22 ↑, prkcbb ↓, mapk10 ↓, gap43 ↓, snap25a ↓, slc17a7 ↑, htt ↓, cplx1 ↓, camk2a ↑, rasgrf1 ↓
Synapsis formation	adam22 ↑, mapk10 ↓, gsk3b ↑, slc6a1b ↑, ache ↑, snap25a ↓, slc17a7 ↑, htt ↓, cplx1 ↓, syn1 ↓, camk2a ↑
Abnormal locomotory behaviour process quality	dyrk1aa ↓, stxbp1a ↑, cyfip2 ↑, ache ↑
IL3 signalling	prkcbb ↓, stat3 ↑, pak1 ↑, sgk3b ↑, slc2a1a ↓
psychological disorder: calcium signalling and adaptive immune system network	snc2b ↓, ppp3ca ↓ ↑, cacna1c ↓, camk2a ↑
**34 °C vs. 26 °C**	
**Networks**	**Molecules**
Synapsis	gabbr2 ↓, gabrg2 ↓, tbc1d24 ↓, stxbp1a ↑, cplx1 ↓, syn1 ↓, git1 ↓, ppp3ca ↑, ppp1r9a ↓, map1b ↑, mapk10 ↓
Regulation of cytoskeleton organization	spns2 ↓, gfap ↑, htt ↓, hsp90aa1.1 ↑, dpysl3 ↑, dync1h1 ↑, ppp1r9a ↓, map1b ↑, tuba1l2 ↓
Establishment of cell polarity	cdh2 ↑, map1b ↑
**KO BDNF and HT BDNF at 26 °C and 34 °C vs. WT**	
**Networks**	**Molecules**
Synapsis	rapgef2 ↓, nptnb ↓, git1 ↓, dcc ↓, pak1 ↑, dnm1 ↓, ctnnd2a ↑, cdk5 ↓ ↑, gsk3b ↓, mapk10 ↓, ppp3ca ↓ ↑, dnm1b ↓, grid2 ↓, map2 ↓ ↑, slc17a7 ↓ ↑, sptbn1 ↓, tbc1d24 ↓, stxbp1a ↓, shank2 ↑, camk2a ↓, syn2a ↓, slc6a1b ↑, tnr ↓ ↑, vamp2 ↑, gap43 ↑, cplx2 ↓ ↑, rab3aa ↓, cplx1 ↓ ↑, fmr1 ↓, mecp2 ↓ ↑, camk4 ↓, rtn4a ↑, cyfip2 ↓
Stress response	sod2 ↓, sod1 ↑, hsp90a1 ↓, capzb ↓, tuba1a ↓, gsk3b ↓, camk2a ↓, mapk1 ↓
Response to heat stress	mapk1 ↓, gsk3b ↓, hsp90ab1 ↓, camk2a ↓

**Table 5:** Top five upstream regulators. The analysis was conducted using IPA on the datasets reported in Supplementary Tables S1–S8. The table presents the top five upstream regulators for each condition. Non-protein factors such as nucleic acids, drugs, chemicals, etc., are highlighted in grey, while transcription regulators are indicated in italics. Ac., activated; In., inhibited.

Gene (Human)	Gene Symbol (*Danio rerio*)	Ensembl Gene ID (*Danio rerio*)	Protein Name	*p*-Value	Predicted Activation	Gene (Human)	Gene Symbol (*Danio rerio*)	Ensembl Gene ID (*Danio rerio*)	Protein Name	*p*-Value	Predicted Activation
ACUTE_18 °C						CHRONIC_18 °C					
*KDM5A*	kdm5a	ENSDARG00000104567	*Lysine Demethylase 5A Transcriptor regulator*	3.80 × 10^−12^		*TP53*	tp53	ENSDARG00000035559	Tumor Protein P53	3.88 × 10^−22^	
*RICTOR*	rictora	ENSDARG00000100867	Rapamycin-Insensitive Companion Of MTOR	4.95 × 10^−12^		*MAPT*	mapta maptb	ENSDARG00000089314; ENSDARG00000087616	Microtubule associated protein tau	3.16 × 10^−18^	
	rictorb	ENSDARG00000002020									
*UQCC3*	uqcc3	ENSDARG00000093382	Ubiquinol-Cytochrome C Reductase Complex Assembly Factor 3	1.30 × 10^−11^		*APP*	appa; appb	ENSDARG00000104279; ENSDARG00000055543	Amyloid Beta Precursor Protein	8.61 × 10^−15^	
*HTT*	Htt	ENSDARG00000052866	Huntingtin	9.49 × 10^−11^		*NFE2L2*	nfe2l2a	ENSDARG00000042824	*NFE2 Like BZIP Transcription Factor 2*	1.25 × 10^−14^	
*MAPT*	mapta	ENSDARG00000089314	Microtubule associated protein tau	2.21 × 10^−10^		1,2-dithiol-3-thione			Chemical Reagent	1.34 × 10^−14^	
	maptb	ENSDARG00000087616									
**ACUTE_34 °C**						**CHRONIC_34 °C**					
*APP*	appa; appb	ENSDARG00000104279; ENSDARG00000055543	Amyloid Beta Precursor Protein	9.84 × 10^−13^		*MAPT*	mapta; maptb	ENSDARG00000089314; ENSDARG00000087616	Microtubule associated protein tau	2.94 × 10^−19^	
CD437			chemical drug	4.69 × 10^−12^		*APP*	appa; appb	ENSDARG00000104279; ENSDARG00000055543	Amyloid Beta Precursor Protein	7.82 × 10^−16^	
*MAPT*	mapta; maptb	ENSDARG00000089314; ENSDARG00000087616	Microtubule associated protein tau	1.12 × 10^−11^		*NFE2L2*	nfe2l2a	ENSDARG00000042824	NFE2 Like BZIP Transcription Factor 2	2.41 × 10^−15^	In.
ST1926			chemical drug	3.58 × 10^−10^		*TP53*	tp53	ENSDARG00000035559	Tumor Protein P53	2.56 × 10^−15^	
*Pln*	pln	ENSDARG00000069404	Phospholamban	6.80 × 10^−10^		1,2-dithiol-3-thione			Chemical Reagent	3.33 × 10^−14^	In.
**KO BDNF_26 °C**						**HT BDNF_26 °C**					
*MAPT*	mapta; maptb	ENSDARG00000089314; ENSDARG00000087616	Microtubule associated protein tau	2.92 × 10^−52^		*MAPT*	mapta; maptb	ENSDARG00000089314; ENSDARG00000087616	Microtubule associated protein tau	1.75 × 10^−19^	
*APP*	appa; appb	ENSDARG00000104279; ENSDARG00000055543	Amyloid Beta Precursor Protein	2.32 × 10^−37^		ST1926			chemical drug	5.68 × 10^−15^	
CD 437			chemical drug	2.58 × 10^−33^	Ac.	CD437			chemical drug	5.11 × 10^−14^	
*PSEN1*	psen1	ENSDARG00000004870	Presenilina 1	4.90 × 10^−32^		*UQCC3*	uqcc3	ENSDARG00000093382	Ubiquinol-Cytochrome C Reductase Complex Assembly Factor 3	1.07 × 10^−13^	
*TP53*	tp53	ENSDARG00000035559	Tumor Protein P53	2.04 × 10^−31^		beta-estradiol			Chemical-endogenous	2.65 × 10^−12^	In.
**KO BDNF_34 °C**						**HT BDNF_34 °C**					
*MAPT*	mapta; maptb	ENSDARG00000089314; ENSDARG00000087616	Microtubule associated protein tau	4.50 × 10^−80^		CD437			Chemical drug	6.84 × 10^−14^	
*CLPP*	clpp	ENSDARG00000020679	Caseinolytic Mitochondrial Matrix Peptidase Proteolytic Subunit	2.98 × 10^−49^	Activated	*MAPT*	mapta; maptb	ENSDARG00000089314; ENSDARG00000087616	Microtubule associated protein tau	1.30 × 10^−13^	
*APP*	appa; appb	ENSDARG00000104279; ENSDARG00000055543	Amyloid Beta Precursor Protein	1.81 × 10^−48^		*RICTOR*	rictora; rictorb	ENSDARG00000100867; ENSDARG00000002020	Rapamycin-Insensitive Companion Of MTOR	7.45 × 10^−13^	
*PSEN1*	psen1	ENSDARG00000004870	Presenilina 1	5.79 × 10^−44^		Sirolimus			Chemical drug	9.20 × 10^−12^	
*UQCC3*	uqcc3	ENSDARG00000093382	Ubiquinol-Cytochrome C Reductase Complex Assembly Factor 3	4.60 × 10^−43^		*TP53*	tp53	ENSDARG00000035559	Tumor protein P53	7.17 × 10^−11^	Ac.

## Data Availability

The mass spectrometry proteomics data have been deposited in the ProteomeXchange Consortium via the PRIDE partner repository with the dataset identifiers PXD009934 [12], PXD016847 [10] and PXD030733 [9].

## Supplementary Materials

The supporting information can be downloaded at https://www.mdpi.com/article/10.3390/ijms242115735/s1.
