# Supplementary material for: The Neurotoxic Effect of Environmental Temperature Variation in Adult Zebrafish (Danio rerio)
Source: Int J Mol Sci. 2023 Oct 29;24(21):15735. doi: 10.3390/ijms242115735 (PMC10648238; doi:10.3390/ijms242115735)
Supplement: Supplementary file 1 [file ijms-24-15735-s001.zip › Tables S11_revised version 28 10 2023.pdf]

		WT_18°C vs. WT_26°C		WT_34°C vs. WT_26°C			HT_26°C vs. WT_26°C	KO_26°C vs. WT_26°C	HT_34°C vs. WT_34°C	KO_34°C vs. WT_34°C
	Acute (A) or Chronic (C) treatment	A	C	A	C	C	C	C	C	C
	Reference	[1]	[2]	[1]	[2]	[3]	[3]	[3]	[3]	[3]
YMT	Time immobile	↑	NA	-	NA	↓	↓	↓	-	-
	Average speed	↓	↓	-	↑	↑	-	↑	-	↑
	Maximum speed	-	NA	-	NA	-	-	-	-	-
	Total distance travelled	↓	↓	-	↑	↑	-	↑	-	↑
	Meandering	-	NA	-	NA	-	-	↓	-	↓
	Reference	[1]		[1]	[4]	[3]	[3]	[3]	[3]	[3]
NTT	Transition	↓	NA	-	↑	↑	-	↑	-	↑
	Transition to top area	↓	NA	-	↑	-	-	↑	-	↑
	Time in top areas	↓	NA	-	↑	-	-	↑	-	↑
	Distance travelled in top area	↓	NA	-	↑	↑	-	↑	-	↑
	Latency to enter the top area	↑	NA	-	↓	↓	-	↓	-	-
LDT	Time spent in dark area	↑	NA	-	↓	↓	↓	↓	-	-
	Transitions	↓	NA	-	-	-	-	-	-	-
SPT	Transitions to social area	↓	NA	-	-	-	-	-	-	↑
	Time spent in social zone	-	NA	-	↓	↓	-	-	-	-
	Distance travelled in social area	-	NA	-	↓	↓	-	-	-	-
MBT	Mirror approach zone entries	↓	NA	-	-	-	-	-	-	-
	Mirror approach zone time spent	-	NA	-	-	-	-	-	-	-
	Mirror bites	-	NA	-	-	↓	↓	↓	-	-
	Mirror biting latency	-	NA	-	↓	-	-	↑	-	-

		Trial 4 vs. trial 1								
		WT_18°C		WT_34°C			HT_26°C	KO_26°	HT_34°C	KO_34°C
	Acute (A) or Chronic (C) treatment	A	C	A	C	C	C	C	C	C
	Reference	[1]	[2]	[1]	[2]	[3]	[3]	[3]	[3]	[3]
YMT	Entries in New arm (%)	-	↑	-	-	-	-	-	-	-
	Time spent in New arm (%)	-	↑	-	-	-	-	-	-	-

Supplementary Table S11. Schematic summary of behavioural analysis of adult (WT, BDNF$^{+/-}$ HT or BDNF$^{-/-}$ KO) zebrafish kept at low (18°C), control (26°C), or high (34°C) temperature for 4 days (acute treatment) or 21 days (chronic treatment) subjected to Y-maze test (YMT), novel tank diving test (NTT), light and dark preference test (LDT), social preference test (SPT) and mirror biting test (MBT) [1-4]. The comparisons WT_18°C vs. WT_26°C (acute and chronic), WT_34°C vs. WT_26°C (acute and chronic), HT_26°C vs. WT_26°C (chronic), KO_26°C vs. WT_26°C (chronic), HT_34°C vs. WT_34°C (chronic), KO_34°C vs. WT_34°C (chronic) are considered. Reduction (↓), increase (↑), not available (NA), not statistically significant difference (-), acute treatment (A), chronic treatment (C).

1 Nonnis, S.; Angiulli, E.; Maffioli, E.; Frabetti, F.; Negri, A.; Cioni, C.; Alleva, E.; Romeo, V.; Tedeschi, G.; Toni, M., Acute environmental temperature variation affects brain protein expression, anxiety and explorative behaviour in adult zebrafish. *Scientific reports* **2021,** 11, (1), 2521.

2 Toni, M.; Angiulli, E.; Miccoli, G.; Cioni, C.; Alleva, E.; Frabetti, F.; Pizzetti, F.; Grassi Scalvini, F.; Nonnis, S.; Negri, A.; Tedeschi, G.; Maffioli, E., Environmental temperature variation affects brain protein expression and cognitive abilities in adult zebrafish (Danio rerio): A proteomic and behavioural study. *Journal of proteomics* **2019,** 204, 103396.

3 Maffioli, E.; Angiulli, E.; Nonnis, S.; Grassi Scalvini, F.; Negri, A.; Tedeschi, G.; Arisi, I.; Frabetti, F.; D'Aniello, S.; Alleva, E.; Cioni, C.; Toni, M., Brain Proteome and Behavioural Analysis in Wild Type, BDNF(+/-) and BDNF(-/-) Adult Zebrafish (Danio rerio) Exposed to Two Different Temperatures. *International journal of molecular sciences* **2022,** 23, (10).

4 Angiulli, E.; Pagliara, V.; Cioni, C.; Frabetti, F.; Pizzetti, F.; Alleva, E.; Toni, M., Increase in environmental temperature affects exploratory behaviour, anxiety and social preference in Danio rerio. *Scientific reports* **2020,** 10, (1), 5385.
